# Supplementary material for: Evidence for a competitive relationship between executive functions and statistical learning
Source: NPJ Sci Learn. 2024 Apr 12;9:30. doi: 10.1038/s41539-024-00243-9 (PMC11014972; doi:10.1038/s41539-024-00243-9)
Supplement: Supplementary file 1 — Supplemenatry Material [file 41539_2024_243_MOESM1_ESM.pdf]

Supplementary material

CCMA using all EF measures

In the main text, we report the results of the CCMA pooling effect sizes of the EF – implicit SL correlations, using only the EF measures that are shared by both studies for the factor scores. We also carried out the same analysis using all EF measures. We obtained highly similar results. We again found very little heterogeneity across the two studies. The between-study heterogeneity variance was estimated at $\tau^2 = 0$, with an I^2 value of 0%, Cochran's Q test was also not statistically significant, Q(1) = 0.02, p = .890. The pooled effect size was negative and significantly different from zero, r = -.162, 95% CI = [-.264, -.057], p = .003 (Supplementary Figure S1).

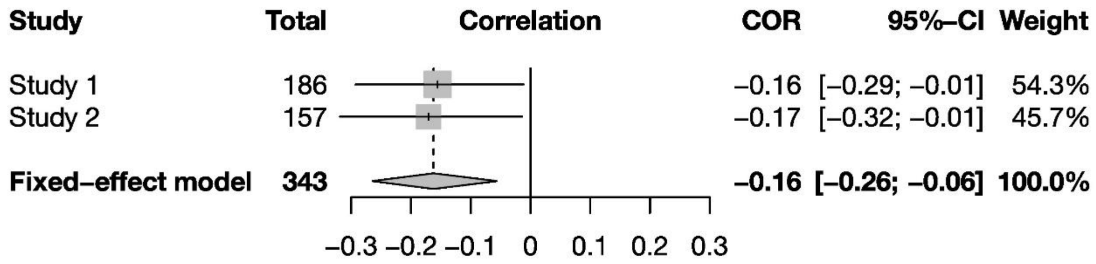

Supplementary Figure 1. CCMA of the two studies. Pearson's r between EF factor scores and implicit SL learning scores, along with its 95% CI is shown next to each individual study. Below, the pooled effect size from a fixed-effect meta-analytic model and its 95% CI is shown. Individual and total sample sizes are also indicated, as well as study weight. Factor scores were derived from all EF tasks.

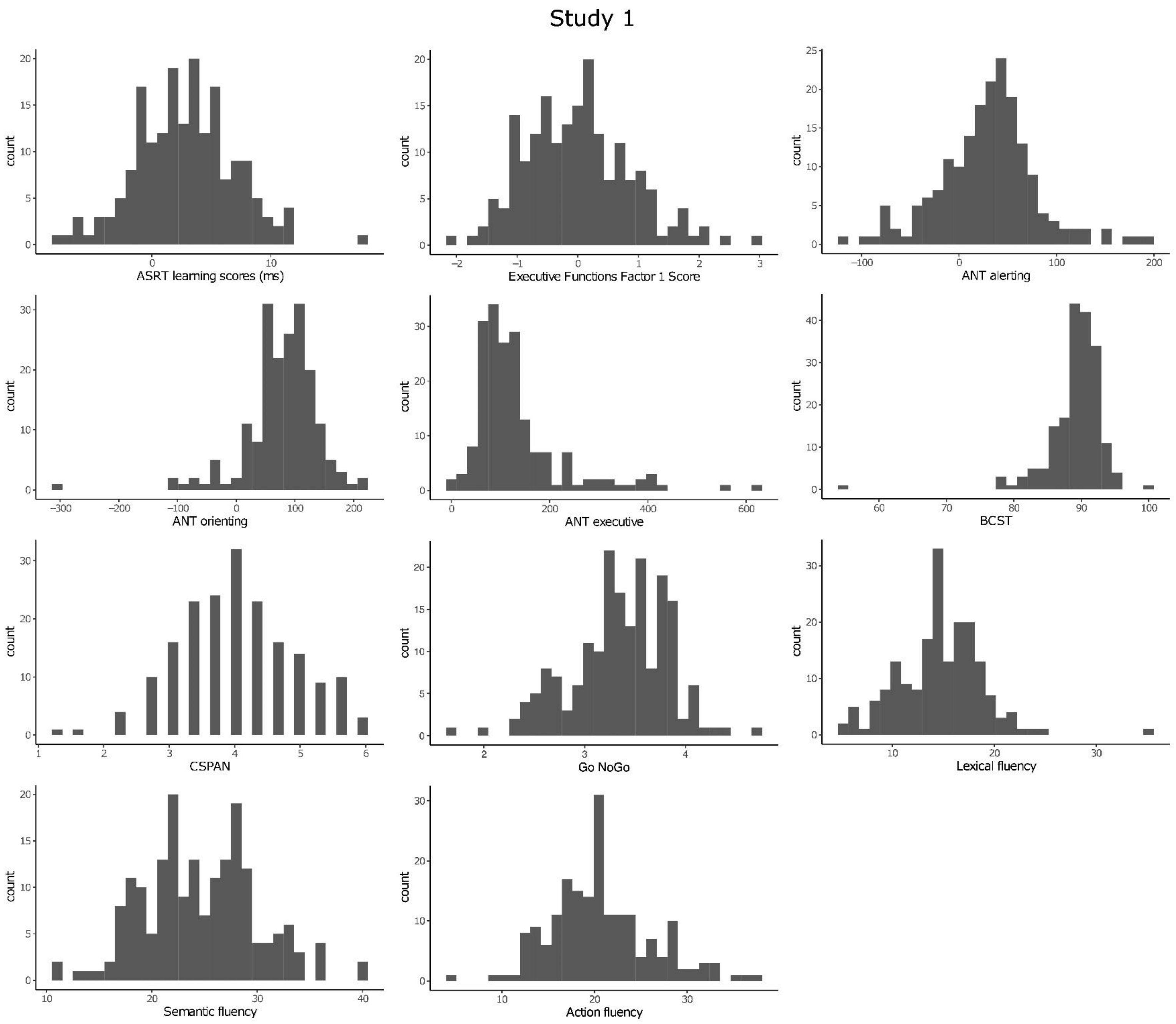

Supplementary Figure 2. Histograms showing the distribution of the main variables of Study 1, including the EF factor score, the ASRT learning scores, and all individual EF task scores.

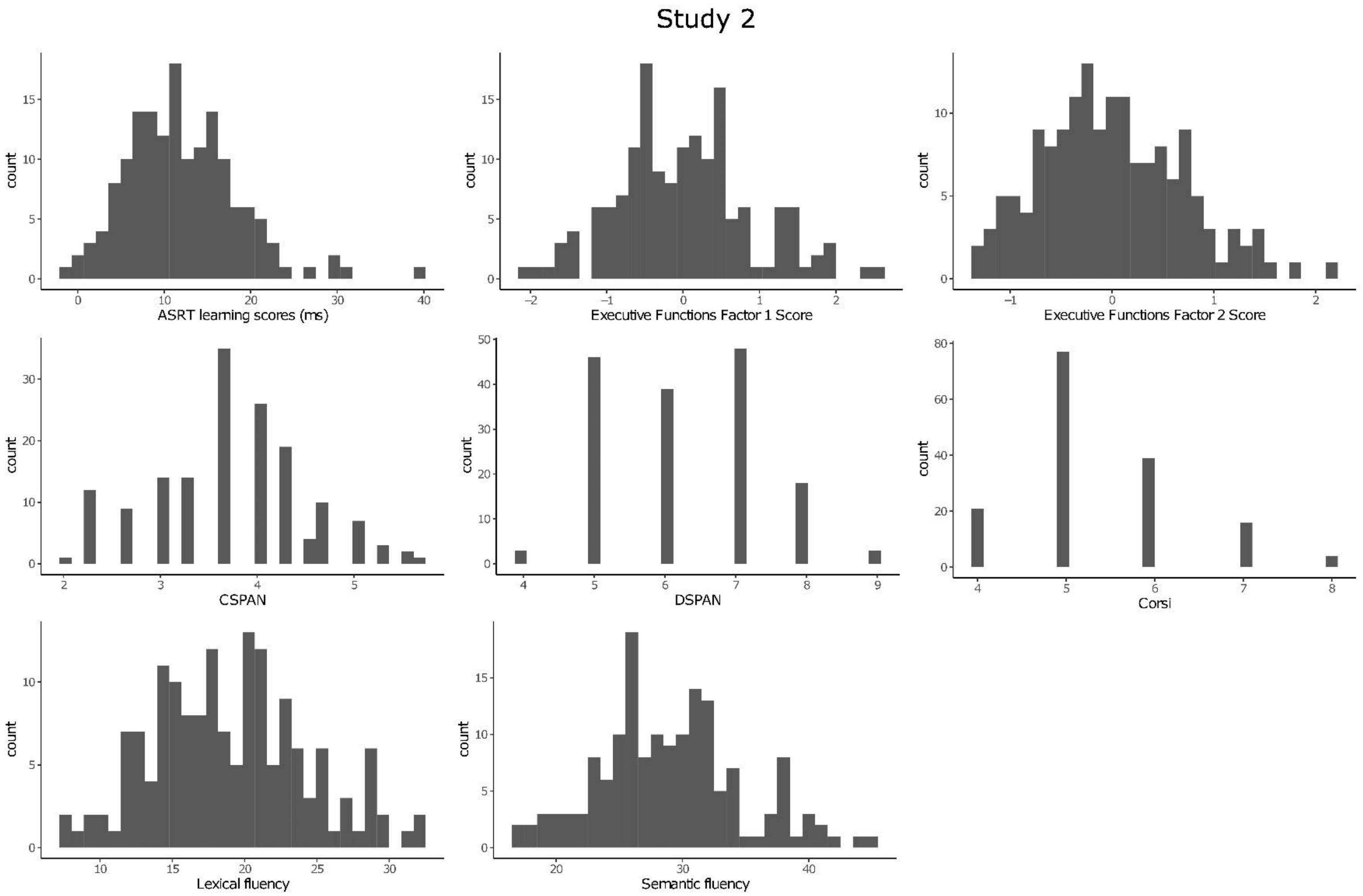

Supplementary Figure 3. Histograms showing the distribution of the main variables of Study 2, including the EF factor scores, the ASRT learning scores, and all individual EF task scores.

**Type III test of effects**	*F*	*df*	*p*
**Triplet Type**	83.53	1, 8294.00	**< .001**
**Block**	40.73	1, 183.97	**< .001**
**EF 1**	7.99	1, 184.01	**.004**
**Triplet Type x Block**	10.24	1, 8924.00	**.001**
**Triplet Type x EF 1**	4.54	1, 8924.00	**.031**
Block x EF 1	0.36	1, 183.97	.548
Triplet Type x Block x EF 1	0.39	1, 8924.00	.535

**Fixed effects**	*b*	*SE b*	*95% CI*	*t*	*df*	*p*
**(Intercept)**	366.79	2.07	362.71 – 370.87	177.47	184.01	**< .001**
**Triplet Type [High]**	-1.42	0.16	-1.73 – -1.12	-9.14	8924.00	**< .001**
**Block**	-0.31	0.05	-0.41 – -0.22	-6.38	183.97	**< .001**
**EF 1**	-6.76	2.39	-11.48 – -2.04	-2.83	184.01	**.005**
**Triplet Type [High] × Block**	-0.07	0.02	-0.11 – -0.03	-3.20	8924.00	**.001**
**Triplet Type [High] × EF 1**	0.39	0.18	0.04 – 0.74	2.16	8924.00	**.031**
Block × EF 1	0.03	0.06	-0.08 – 0.15	0.60	183.97	.548

Triplet Type [High] × Block × EF 1	0.02	0.03	-0.03 – 0.06	0.62	8924.00	.535
**Random Effects**						
σ^2	225.95					
$\tau_{00\ \text{Subject}}$	790.01					
$\tau_{11\ \text{Subject.Block}}$	0.36					
$\rho_{01\ \text{Subject}}$	-0.18					
ICC	0.78					
N_{Subject}	186					
Observations	9300					
Marginal R^2 / Conditional R^2	0.039 / 0.790					

Supplementary Table 1. Top table shows the Type 3 tests of fixed effects. Bottom table shows regression coefficients of fixed effects and summary information about the random effects. The marginal R-squared considers only the variance of the fixed effects, while the conditional R-squared takes both the fixed and random effects into account (based on Nakagawa et al., 2017). Degrees of freedom are based on Satterthwaite's approximation. Significant terms are highlighted in bold. Terms in brackets indicate the levels of the factors that are contrasted against the reference level.

Model equation in *lmer* syntax: RT ~ EF1*Block*TripletType + (Block | Subject)

36

**Type III test of effects**	*F*	*df*	*p*
**Triplet Type**	1289.09	1, 13810.00	**< .001**
**Block**	662.76	1, 154.00	**< .001**
EF 1	2.93	1, 154.02	.089
**EF 2**	9.67	1, 154.02	**.002**
**Triplet Type x Block**	156.70	1, 13810.00	**< .001**
**Triplet Type x EF 1**	5.26	1, 13810.00	**.022**
Triplet Type x EF 2	0.13	1, 13810.00	.723
**Block x EF 1**	8.31	1, 154.00	**.005**
Block x EF 2	2.95	1, 154.00	.088
Triplet Type x Block x EF 1	0.13	1, 13810.00	.717
Triplet Type x Block x EF 2	0.89	1, 13810.00	.347

37

**Fixed effects**	*b*	*SE b*	*95% CI*	*t*	*df*	*p*
**(Intercept)**	377.48	2.19	373.16 – 381.80	172.59	154.02	**< .001**
**Triplet Type [High]**	-6.25	0.17	-6.59 – -5.91	-35.90	13810.00	**< .001**
**Block**	-1.12	0.04	-1.20 – -1.03	-25.74	154.00	**< .001**
EF 1	-4.28	2.50	-9.22 – 0.66	-1.71	154.02	.089
**EF 2**	-9.84	3.16	-16.08 – -3.59	-3.11	154.02	**.002**

**Triplet Type [High] × Block**	-0.17	0.01	-0.19 – -0.14	-12.52	13810.00	**< .001**
**Triplet Type [High] × EF 1**	0.46	0.20	0.07 – 0.85	2.29	13810.00	**.022**
Triplet Type [High] × EF 2	0.09	0.25	-0.40 – 0.58	0.35	13810.00	.723
**Block × EF 1**	0.14	0.05	0.04 – 0.24	2.88	154.00	**.005**
Block × EF 2	0.11	0.06	-0.02 – 0.23	1.72	154.00	.088
Triplet Type [High] × Block × EF 1	0.01	0.02	-0.02 – 0.04	0.36	13810.00	.717
Triplet Type [High] × Block × EF 2	-0.02	0.02	-0.06 – 0.02	-0.94	13810.00	.347
**Random Effects**						
σ^2	428.08					
$\tau_{00\ Subject}$	746.25					
$\tau_{11\ Subject.Block}$	0.27					
$\rho_{01\ Subject}$	-0.27					
ICC	0.65					
$N_{Subject}$	157					
Observations	14130					

Marginal R^2 / Conditional R^2	0.210 / 0.722

Supplementary Table 2. Top table shows the Type 3 tests of fixed effects. Bottom table shows regression coefficients of fixed effects and summary information about the random effects. The marginal R-squared considers only the variance of the fixed effects, while the conditional R-squared takes both the fixed and random effects into account (based on Nakagawa et al., 2017). Degrees of freedom are based on Satterthwaite's approximation. Significant terms are highlighted in bold. Terms in brackets indicate the levels of the factors that are contrasted against the reference level.

Model equation in *lmer* syntax: RT ~ (EF1+EF2)*Block*TripletType + (Block | Subject)
