# Supplementary material for: Evidence for a competitive relationship between executive functions and statistical learning
Source: NPJ Sci Learn. 2024 Apr 12;9:30. doi: 10.1038/s41539-024-00243-9 (PMC11014972; doi:10.1038/s41539-024-00243-9)
Supplement: Supplementary file 2 — nr-reporting-summary.pdf [file 41539_2024_243_MOESM2_ESM.pdf]

nature portfolio

Corresponding author(s): Dezso Nemeth

Last updated by author(s): Mar 28, 2024

Reporting Summary

Nature Portfolio wishes to improve the reproducibility of the work that we publish. This form provides structure for consistency and transparency in reporting. For further information on Nature Portfolio policies, see our Editorial Policies and the Editorial Policy Checklist.

Statistics

For all statistical analyses, confirm that the following items are present in the figure legend, table legend, main text, or Methods section.

n/a	Confirmed	
☐	☒	The exact sample size (*n*) for each experimental group/condition, given as a discrete number and unit of measurement
☐	☒	A statement on whether measurements were taken from distinct samples or whether the same sample was measured repeatedly
☐	☒	The statistical test(s) used AND whether they are one- or two-sided<br>*Only common tests should be described solely by name; describe more complex techniques in the Methods section.*
☐	☒	A description of all covariates tested
☐	☒	A description of any assumptions or corrections, such as tests of normality and adjustment for multiple comparisons
☐	☒	A full description of the statistical parameters including central tendency (e.g. means) or other basic estimates (e.g. regression coefficient) AND variation (e.g. standard deviation) or associated estimates of uncertainty (e.g. confidence intervals)
☐	☒	For null hypothesis testing, the test statistic (e.g. *F*, *t*, *r*) with confidence intervals, effect sizes, degrees of freedom and *P* value noted<br>*Give P values as exact values whenever suitable.*
☐	☒	For Bayesian analysis, information on the choice of priors and Markov chain Monte Carlo settings
☒	☐	For hierarchical and complex designs, identification of the appropriate level for tests and full reporting of outcomes
☐	☒	Estimates of effect sizes (e.g. Cohen's *d*, Pearson's *r*), indicating how they were calculated

Our web collection on statistics for biologists contains articles on many of the points above.

Software and code

Policy information about availability of computer code

Data collection	The ASRT task were coded in Presentation® 21.1.<br>We used the BCST and GNG tasks from the PEBL software.<br>The ANT and CSPAN tasks were coded in E-prime 3.0.
Data analysis	Data analysis was carried in RStudio version 2023.6.2.561.

For manuscripts utilizing custom algorithms or software that are central to the research but not yet described in published literature, software must be made available to editors and reviewers. We strongly encourage code deposition in a community repository (e.g. GitHub). See the Nature Portfolio guidelines for submitting code & software for further information.

Data

Policy information about availability of data

All manuscripts must include a data availability statement. This statement should provide the following information, where applicable:

- Accession codes, unique identifiers, or web links for publicly available datasets
- A description of any restrictions on data availability
- For clinical datasets or third party data, please ensure that the statement adheres to our policy

All data necessary to replicate the findings of this manuscript can be found on the OSF platform, at the following link: https://osf.io/2asnb/

Research involving human participants, their data, or biological material

Policy information about studies with human participants or human data. See also policy information about sex, gender (identity/presentation), and sexual orientation and race, ethnicity and racism.

Reporting on sex and gender	Study 1 comprises 186 participants (108 females).<br>Study 2 comprises 157 participants (133 females).
Reporting on race, ethnicity, or other socially relevant groupings	NA
Population characteristics	Study 1 comprises 186 participants (108 females). Age in years (Mean; SD) = 22.06; 2.78<br>Study 2 comprises 157 participants (133 females). Age in years (Mean; SD) = 21.80; 4.33
Recruitment	For Study 1 participants were recruited through online advertisement.<br>For Study 2 participants were recruited at Eötvös Loránd University, Budapest, Hungary.
Ethics oversight	The "Comité de Protection des Personnes, CPP Est I" gave ethical approval for Study 1.<br>Study 2 was approved by the United Ethical Review Committee for Research in Psychology (EPKEB) in Hungary (Approval number: 30/2012) and by the research ethics committee of Eötvös Loránd University, Budapest, Hungary.

Note that full information on the approval of the study protocol must also be provided in the manuscript.

Field-specific reporting

Please select the one below that is the best fit for your research. If you are not sure, read the appropriate sections before making your selection.

☐ Life sciences ☒ Behavioural & social sciences ☐ Ecological, evolutionary & environmental sciences

For a reference copy of the document with all sections, see nature.com/documents/nr-reporting-summary-flat.pdf

Behavioural & social sciences study design

All studies must disclose on these points even when the disclosure is negative.

Study description	This study presents a thorough investigation into the association between implicit statistical learning (SL) and executive function (EF) through the integration of multiple datasets and the analysis of large participant cohorts. To assess SL and EF, quantitative measures derived from reliable and well-established neuropsychological tasks were utilized.
Research sample	Study 1 involved 186 participants (108 females) with an average age of 22.06 years (SD = 2.78). These participants were healthy young adults recruited from the general public in France, all under the age of 35.<br>Study 2 included 157 participants (133 females) with an average age of 21.80 years (SD = 4.33). These individuals were university students from Hungary.<br>Given our primary aim to investigate the association between implicit statistical learning and executive functions in neurotypical adults, these two samples serve as suitable representatives of the broader adult population.
Sampling strategy	The relationship between SL and EF has been investigated in two prior studies, albeit with relatively small sample sizes of 22 and 40 participants, respectively. This limited sample size resulted in low statistical power, hindering the establishment of reliable correlations between the tasks. Moreover, the absence of a replication sample in either study leaves the robustness of their findings uncertain. Following recommendations by Schönbrodt & Perugini (2013), we aimed to address these limitations by defining a sample size larger than 250 participants across two combined studies, thus providing internal replication.
Data collection	This study encompasses a diverse array of tasks, all of which, except for the verbal fluency tasks administered via pen and paper, were computerized.
Timing	Both studies were conducted across two sessions. The first session lasted approximately 2 hours, while the second session lasted approximately 1 hour. These sessions were scheduled on different dates, and the gap between each session varied according to each participant's availability.
Data exclusions	For Study 1, initially, 189 participants were recruited. However, two participants did not return for the second session, and one participant did not comply with the task instructions during the first session. As a result, three participants were excluded from the final analysis.<br><br>In Study 2, initially, 180 participants were recruited. Among these participants, 23 had missing data in one of the tasks during the second session and were consequently excluded from the final analyses.
Non-participation	2 participants dropped out of the experiment.

Randomization	Participants were not allocated into different groups.

Reporting for specific materials, systems and methods

We require information from authors about some types of materials, experimental systems and methods used in many studies. Here, indicate whether each material, system or method listed is relevant to your study. If you are not sure if a list item applies to your research, read the appropriate section before selecting a response.

Materials & experimental systems

n/a	Involved in the study
☒	☐ Antibodies
☒	☐ Eukaryotic cell lines
☒	☐ Palaeontology and archaeology
☒	☐ Animals and other organisms
☒	☐ Clinical data
☒	☐ Dual use research of concern
☒	☐ Plants

Methods

n/a	Involved in the study
☒	☐ ChIP-seq
☒	☐ Flow cytometry
☒	☐ MRI-based neuroimaging

Plants

Seed stocks	NA
Novel plant genotypes	NA
Authentication	NA
